# Changes in circulating microRNA after recumbent isometric yoga practice by patients with myalgic encephalomyelitis/chronic fatigue syndrome: an explorative pilot study

**DOI:** 10.1186/s13030-019-0171-2

**Published:** 2019-12-02

**Authors:** Shu Takakura, Takakazu Oka, Nobuyuki Sudo

**Affiliations:** 1 Department of Psychosomatic Medicine, Kyushu University Hospital, Maidashi 3-1-1, Higashi-ku, Fukuoka, 812-8582 Japan; 2 Department of Psychosomatic Medicine, International University of Health and Welfare Hospital, Iguchi 537-3, Nasushiobara-shi, Tochigi-ken 329-2763 Japan; 3 Department of Psychosomatic Medicine, Graduate School of Medical Sciences, Kyushu University, Maidashi 3-1-1, Higashi-ku, Fukuoka, 812-8582 Japan

**Keywords:** Chronic fatigue syndrome (CSF), Isometric yoga, Fatigue, microRNA (miRNA), Myalgic encephalomyelitis (ME)

## Abstract

**Background:**

Yoga is a representative mind-body therapy. Our previous studies have demonstrated that isometric yoga (i.e. yoga programs that we developed so individuals can practice yoga poses with a self-adjustable isometric load) reduces the fatigue of patients with myalgic encephalomyelitis/chronic fatigue syndrome (ME/CFS); however, the underlying mechanisms remain unclear. Several studies have suggested that the micro-ribonucleic acid (miRNA) expression of ME/CFS patients is different from that of healthy subjects. However, it has not to date been determined if the practice of isometric yoga can affect miRNA expression. Therefore, we sought to investigate if isometric yoga is associated with changes in the expression levels of serum miRNA of patients with ME/CFS.

**Methods:**

The study included nine patients with ME/CFS who failed to show satisfactory improvement after at least 6 months of treatment administered at our hospital. Patients practiced recumbent isometric yoga for 3 months; they met with a yoga instructor every 2 to 4 weeks and participated in daily in-home sessions. The effect of recumbent isometric yoga on fatigue was assessed by comparing pre- and post-intervention scores on the Japanese version of the 11-item Chalder fatigue scale (CFQ 11). Patient blood samples were drawn pre- and post-intervention, just prior to practicing recumbent isometric yoga with an instructor. The serum was used for miRNA array analysis with known human miRNAs.

**Results:**

The average CFQ 11 score decreased significantly (from 25.3 ± 5.5 to 17.0 ± 5.8, *p* <  0.0001) after practicing recumbent isometric yoga for 3 months. The miRNA microarray analysis revealed that four miRNAs were significantly upregulated, and 42 were downregulated after the intervention period.

**Conclusions:**

This explorative pilot study is the first to demonstrate changes in the serum levels of several miRNAs after regular practice of recumbent isometric yoga. These miRNAs might represent biomarkers for the fatigue-relieving effects of isometric yoga of patients with ME/CFS.

**Trial registration:**

University Hospital Medical Information Network (UMIN CTR) 000023472. Registered Aug 4, 2016.

## Background

Myalgic encephalomyelitis/chronic fatigue syndrome (ME/CFS) is a debilitating disease characterized by persistent or relapsing unexplained fatigue of at least 6 months’ duration that is not relieved by rest and that causes a substantial reduction in daily activities [1, 2]. Currently, the number of effective treatment options for ME/CFS is limited. Therefore, it is important for patients to engage in self-help strategies, such as pacing, to manage their symptoms.

Yoga is a representative mind-body therapy [3] that has been recommended as a self-help tool for coping with ME/CFS [2]. However, there was limited evidence on its efficacy [4] until we investigated the effects of isometric yoga on patients with ME/CFS [5, 6]. Isometric yoga is a yoga program that was developed by Oka et al. specifically for patients with ME/CFS. To date, we have developed two types of isometric yoga programs, enabling patients to practice poses with a self-adjustable isometric load in a seated [5] or recumbent [6] position. The recumbent isometric yoga program was designed for patients who cannot sit for a long period of time and who were reluctant to practice a seated isometric yoga program. We demonstrated that regular practice of both seated [5] and recumbent [6] isometric yoga reduced the fatigue of these patients, according to subjective self-reported measures.

The reason for developing these isometric yoga programs was based on findings that showed the positive effects of yoga on breast cancer patients and survivors. Although these yoga programs differed from that used in our studies, the combined results demonstrated that regular yoga practice can reduce fatigue and improve psychological health outcomes, e.g. reduction of perceived stress, anxiety, depression, and insomnia (for a review, see [7, 8]). This suggested that these yoga-induced effects might also be beneficial for patients with ME/CFS. However, based on our clinical experience, it seemed that patients with ME/CFS would have difficulty practicing yoga programs similar to those designed for patients with breast cancer or healthy individuals. In fact, some patients actually reported experiencing exacerbated fatigue and pain after attending a public yoga class and attempting the same poses as healthy participants. Therefore, we discussed these issues with yoga instructors of the Japan Yoga Therapy Society and with them developed two isometric yoga programs for patients with ME/CFS, taking into consideration the pathophysiological characteristics of the disease and the limitations of the patients. First, given that the patients have severe fatigue and their condition changes moment to moment, we selected easy isometric poses so that patients could adjust the muscular strength they apply depending on their present condition. Second, as patients have orthostatic intolerance, we intentionally did not include any standing postures. Third, given that the patients have severe pain, we avoided poses that would require flexibility and active stretching. Fourth, given that the patients have impaired concentration and short-term memory, we kept the programs as simple as possible. Fifth, because some patients are deconditioned, we designed the programs to help in the prevention of disuse muscular atrophy by including isometric loading. Most importantly, because the patients experience post-exertional malaise, we also designed the programs to enhance the awareness of inner sensations (interoception and proprioception), so that the patients can be more sensitive and aware of the thresholds that induce post-exertional malaise and to keep their daily activities within their personal limits. Furthermore, we asked the yoga instructor to pay careful attention to the patient’s physical condition to avoid exacerbation of symptoms or post-exertion malaise and permitted the instructor to divide and modify the program if necessary. We also asked the instructor to adjust the brightness of ceiling lights and to not play music, use perfume, or touch participants when giving instruction because they may have hyper-sensitivity to light, sound, or smell, and may have allodynia. Given that the severity of symptoms differed from person to person, the yoga instruction was not performed in a group setting but instead on a one-to-one basis. It should be noted that this yoga program was not developed as another option for graded exercise therapy but rather as an exercise to induce post-isometric relaxation [9, 10] and to facilitate awareness for more efficient pacing.

With this newly developed program, we sought to investigate the underlying mechanisms for the fatigue-improving effects of isometric yoga. While the pathophysiological mechanisms of ME/CFS are not yet fully understood, previous studies have suggested several abnormalities in patients with ME/CFS, including dysfunction of the autonomic nervous system, hypothalamic-pituitary-adrenocortical (HPA) axis, and immune system [11]. Autonomic dysfunction is characterized by low vagal tone and sympathetic overactivity [12, 13]. Dysfunctions of the HPA axis include attenuated diurnal changes in cortisol [14] and decreased levels of dehydroepiandrosterone sulfate (DHEA-S) [15]. Dysfunctions of the immune system include increased blood levels of transforming growth factor (TGF)-β1 [16] and proinflammatory cytokines such as tumor necrosis factor (TNF)-α [17, 18]. In our previous study, we demonstrated that seated isometric yoga resulted in improvements in some of these abnormalities, including increased DHEA-S serum levels and the high frequency component of heart rate variability, suggesting an increase in parasympathetic function,and reduced cortisol and TNF-α serum levels in patients with ME/CFS [19]. Therefore, these changes might be associated with the therapeutic mechanisms of isometric yoga on ME/CFS. However, recent findings on the biological abnormalities of patients with ME/CFS, such as peripheral cytokines ([20]; for review, see [21, 22]); are not necessarily consistent. Thus, caution should be taken when interpreting the role of isometric yoga on inducing these fatigue-relieving effects.

To further investigate the mechanisms behind the fatigue-relieving effects of practicing isometric yoga, we extended our study to assess changes in a new class of biomarkers, microribonucleic acids (miRNAs). Recent studies have suggested that miRNAs are involved in ME/CFS [23–25]. miRNAs are single-stranded, non-coding small RNAs, 18–25 nucleotides in length, that bind to the 3’UTRs of target messenger RNAs (mRNAs) and contribute to gene silencing by suppressing protein production or facilitating mRNA degradation [26, 27]. miRNAs play a critical role in various biological processes, such as cell differentiation, development and homeostasis [28–30], and circulating miRNAs have been proposed as biomarkers for some medical conditions [31–33]. Several studies have demonstrated differences between patients with ME/CFS and non-fatigued control subjects in the levels of miRNA expression in peripheral blood mononuclear cells [25], cytotoxic lymphocytes [23], and plasma [24]. These studies proposed the use of miRNAs as potential biomarkers of ME/CFS [24, 25].

To date, however, it is not known if yoga affects the miRNA expression of patients with ME/CFS. Therefore, we sought to undertake this exploratory pilot study to investigate if the regular practice of isometric yoga affects the serum miRNA expression of patients with ME/CFS.

## Methods

This study was approved by the Institutional Review Board of Kyushu University. Written informed consent was obtained from all study participants before they were enrolled.

### Subjects

This study included nine female patients with ME/CFS. They were all outpatients who visited the Department of Psychosomatic Medicine of Kyushu University Hospital and who failed to show satisfactory improvement after at least 6 months of other therapies administered at our hospital. None of the patients had participated in our previous study [6]. Within our department, ME/CFS patients are initially treated by providing guidance on self-help strategies so they can engage in appropriate coping, self-monitoring of disrupted diurnal rhythms of body temperature and its modification, psychotherapy to reduce guilt, self-blame, anxiety, tension, rumination of negative thoughts, and/or environmental arrangements at home, the workplace or school, as well as pharmacotherapy [34, 35]. Treatments are chosen based on the severity of symptoms, the evaluation of a patient’s acceptance of this disease and adaptive transition for more appropriate coping, and the patient’s motivation or preference for the treatments. For example, if a patient asks to not be prescribed any medication, they are treated by only non-pharmacological means. After undergoing treatment in our department for more than 6 months, if the patient does not feel like they achieved satisfactory improvement and asks for further treatment, we propose recumbent isometric yoga as an additional treatment option because our previous study demonstrated that the regular practice of recumbent isometric yoga reduced fatigue [6]. Among the patients who were prescribed isometric yoga, subjects who satisfied the following criteria were enrolled in this study: (1) age between 20 and 70 years; (2) severity of fatigue was serious enough to cause an absence from school or the work place for at least several days per week, but not serious enough to require assistance with activities of daily living; (3) the subject could fill out the questionnaires without assistance; (4) the subject was able to visit the hospital every 2 or 4 weeks; and (5) subjects had not practiced any kind of yoga before, including seated [5, 19] or recumbent [6] isometric yoga.

A diagnosis of ME/CFS was made when the patient met the following diagnostic criteria: the 1994 Fukuda case definition of CFS [1], the 2005 Canadian clinical case definition of ME/CFS [2], the 2011 International Consensus Criteria for ME [36], and the 2015 diagnostic criteria for systemic exertion intolerance disease [37].

### Recumbent isometric yoga program

In the current study, we only used the recumbent isometric program because in a previous study patients with ME/CFS preferred the recumbent isometric yoga program to a seated program, and even patients who had difficulty practicing seated isometric yoga could practice recumbent isometric yoga [6]. The details of the yoga program are described elsewhere [6].

**Fig. 1:**
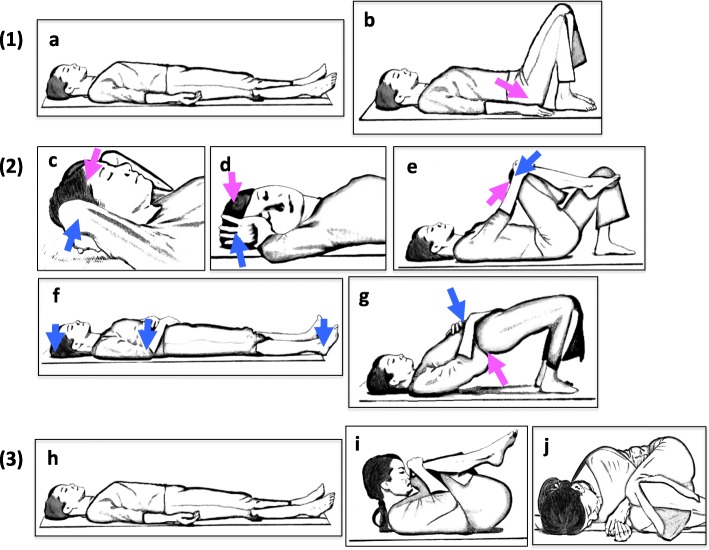
Illustrations of poses used in the recumbent isometric yoga program for ME/CFS. The recumbent isometric yoga program consisted of three parts: (1) Adjusting external and internal conditions. Awareness of body and breathing in a supine position (**a**). Relaxation of excessive lumbar lordosis (**b**). (2) Isometric yoga poses. Isometric yoga for the neck (**c**), the neck and shoulder (**d**), lower back and hips (**e**), and the heels, elbows, and head (**f**). Loading and unloading of the hips (**g**). (3) Deep relaxation and awakening. Sava-asana (**h**). Fetal pose (**i**). Relaxation in the lateral decubitus position (**j**). (modified from Fig. 1 of [6], For a video of this program, watch [38])

The recumbent isometric yoga program was designed so that patients can practice while in bed. It consists of three portions: (1) adjusting external and internal conditions, (2) isometric yoga poses, and (3) deep relaxation, awakening, and generalization of this state to daily life. It requires approximately 20–30 min to complete. However, the length of practice or the number of repetitions of each pose was modified depending on the patient’s condition and severity. (1) Adjusting external and internal conditions: Before starting, the yoga instructor is asked to pay attention to external stimuli such as temperature, humidity, sound, smell, and light, so that patients can practice isometric yoga with minimal stress. During the session, the instructor is also asked to be mindful of the volume and tone of her voice and speed of instruction. At home, patients are asked to be mindful of these factors as well. The patients are then instructed to be aware of their own bodies and natural breathing in a supine relaxing position with their arms and legs spread and eyes closed, i.e. Sava-asana pose (Fig. 1a). After that, the patients are instructed to adjust excessive lumber lordosis so they can feel their waist and back become more relaxed and breathe deeper. This is done by first bending their knees, then placing their hands on the side of their waist and lifting the hips and buttocks slightly. The hips are then slid down towards the heels (Fig. 1b). (2) Isometric yoga poses: Patients practice five isometric yoga poses, as shown in Fig. 1. These poses include isometric loading of the nape of the neck by pushing with both hands (Fig. 1c), isometric loading by rotating the neck to the right and left (Fig. 1d), isometric loading of the lower back and hips (Fig. 1e), isometric loading of the heels, elbows, and head (Fig. 1f), and loading and unloading of the hips (Fig. 1g). These poses are to be performed slowly. During isometric loading, three repetitions are performed at 30–50% of maximum muscle strength while exhaling and saying “umm” twice and silently once. After the isometric loading and while inhaling, patients should relax their muscles while maintaining their position. Once completely relaxed, a return to the first position is required while exhaling slowly, followed by the adoption of Sava-asana. The amount of force, number of repetitions, and break between repetitions should be adjusted for the degree of fatigue experienced by the patient. (3) Deep relaxation, awakening and generalization of this state to daily life: After practicing the series of isometric poses, the patients are asked to focus on relaxed and calm feelings by Sava-asana (Fig. 1h), fetal pose (Fig. 1i), and in the lateral decubitus position (Fig. 1j). After awakening, patients are asked to generalize these feelings and mindset to their daily lives. Thus, the ultimate goal of this program is to make this practice a habit and to nurture a “therapist self”, so that patients can help themselves in a more therapeutic way. This enables patients to be more sensitive to the threshold at which their symptoms become exacerbated or post-exertional malaise is induced and helps them judge wisely how to behave in consideration of their condition, which changes from moment to moment, enabling them to live a daily life with less rumination on idling thoughts and negative feelings.

Isometric yoga differs from traditional yoga postures in several ways. The predominant difference is that the poses consist mainly of isometric muscle contractions, and patients can modulate resistance depending on their condition. The isometric yoga poses do not include isotonic muscular contractions or active stretching and require less physical flexibility, which helps the patient avoid over-stretching and to practice without exacerbation of pain. Furthermore, although isometric loading is a resistance exercise, it also induces post-isometric relaxation, which allows patients to feel relaxed and reduces pain after practice [9, 10]. Another important difference is that the isometric yoga program is not merely a physical exercise but rather an exercise to nurture a “therapist self” for better coping. However, similar to traditional yoga poses, isometric yoga poses are performed slowly with a mindfulness of one’s breath while synchronizing breathing and movement, as well as maintaining an awareness of inner sensations.

### Recumbent isometric yoga intervention

Patients practiced the recumbent isometric yoga program for 3 months. On days when the patients visited the hospital, they practiced on a one-on-one basis with a yoga instructor. In addition to receiving a private lesson, they also practiced this program at home, if they could, with the aid of a digital videodisk, a video (YouTube [38]) or a booklet (the Japanese version is downloadable [39]) on the recumbent isometric yoga program. They also kept a “yoga diary” through which the yoga instructor could monitor the amount of time they practiced and any difficulties or questions they had pertaining to this program.

The instructor had more than 30 years of experience as a yoga instructor and had more than 4 years of experience instructing isometric yoga to patients with ME/CFS (approximately 50 patients in total). During instruction of the isometric yoga program, the instructor paid special attention to the external environment as well as the patient’s physical or psychological conditions. She also answered questions raised by patients. The length of breathing or the number of repetitions of each pose was changed depending on the patient’s condition. If the patient practiced this program with an inappropriate force or breathing pattern or the patient felt this program was difficult, the instructor modified it so that they could practice it in a more comfortable way. With these instructions, no one experienced worsening of symptoms or post-exertional malaise and, even those who did not feel any benefit from this program in the beginning, eventually felt reduction of fatigue by the last session with the instructor.

During the intervention period, any medications that had been previously prescribed were continued and medication dosages were not changed. These drugs included antidepressants, hypnotics, anti-histaminergic drugs, tandospirone [40, 41], midodrine, pregabalin, and Japanese traditional herbal medicines, e.g. hochuekkito, which is often prescribed to improve energy for (Qi)-deficient conditions [42, 43]. No patient was treated with hydrocortisone.

### Measures

At every hospital visit, the patient first had a medical check-up with their physician prior to practicing recumbent isometric yoga with the instructor. On the first and last days of the intervention period, after medical check-up, the patients were allowed a sufficient resting period for at least 30 min before filling out the questionnaire, and blood was drawn from the forearm vein between 2 p.m. and 4 p.m. Administration of the questionnaire and blood sampling were postponed for 2 weeks for one patient who reported having a cold. Serum samples were centrifuged then stored at − 80 °C until measurements were performed.

#### Chalder fatigue scale scoring

The effects of isometric yoga on fatigue were assessed using the Japanese version of the 11-item Chalder FS (CFQ 11) score [44, 45], which is a well-validated, self-reported Likert scale that measures the severity of fatigue of patients with ME/CFS. Scores were obtained on the patient’s first visit to our hospital, then before and after the 3-month intervention period. The maximal score of the CFQ 11 is 33. A nurse who was not otherwise involved in the study administered the questionnaire.

#### RNA extraction and miRNA expression profiling

Total miRNA was extracted from 300 mL of serum using the 3D-Gene® RNA extraction reagent (Toray, Kamakura, Japan). Extracted total RNA was checked using a Bioanalyzer instrument (Agilent, CA, USA) and labeled with the 3D-Gene® miRNA labeling kit (Toray, Kamakura, Japan). Half volumes of labeled RNAs were hybridized onto the 3D-Gene® serum miRNA Oligo chip (Toray, Kamakura, Japan). The annotation and oligonucleotide sequences of the probes conformed to the miRBase miRNA database (http://www.mirbase.org). After stringent washes, fluorescent signals were scanned with the 3D-Gene® Scanner (Toray Industries) and analyzed using 3D-Gene® Extraction software (Toray Industries). The raw data for each spot were normalized by substitution with the mean intensity of the background signal determined by the signal intensities of blank spots with a 95% confidence interval. Measurements of spots with signal intensities greater than two standard deviations (SDs) from the background signal intensity were considered to be valid. The relative expression level of a given miRNA was calculated by comparing the signal intensities of the valid spots throughout the microarray experiments. The normalized data were globally normalized per array, such that the median signal intensity was adjusted to 25.

### Statistical analysis

The normality of the data distribution was evaluated using the Shapiro-Wilk test. Because all data sets showed a normal distribution, a paired-sample *t* test was used to analyze parameters before and after the yoga session. There was no correction for multiple comparisons. All tests were considered statistically significant at *p* <  0.05 and all tests were two-tailed. Data are presented as the means ± standard deviation (SD). All statistical analyses were performed using JMP® for MacOS version 13.

## Results

### Clinical characteristics

Table 1 shows the clinical characteristics of the nine patients included in this study. They were all female, with a mean age of 37.2 ± 9.9 (Table 1). All of the patients were unable to carry on normal activity, were on sick leave if they had been employed, and required rest at home. However, their condition was not so serious that they required assistance in daily living and would be prevented from visiting the hospital. According to the yoga diaries, all participants practiced recumbent isometric yoga at least four times per week. They visited the hospital once a month. The frequency of visits was similar before and after the intervention, except for two patients who visited twice a month just after the start of the intervention to further practice the recumbent isometric yoga with an instructor.

**Table 1:** Patient Characteristics

Patient No.	Age	Sex	CFQ 11 score		
			1st	Before	After
1	34	F	30	31	19
2	26	F	25	27	19
3	40	F	20	28	22
4	37	F	32	32	25
5	32	F	28	16	5
6	61	F	29	28	17
7	32	F	28	24	13
8	39	F	24	24	19
9	34	F	22	18	14

1st: first visit to the hospital

Before: just before starting recumbent isometric yoga

After: 3 months after practicing recumbent isometric yoga

### Changes in fatigue after practicing recumbent isometric yoga

The CFQ 11 scores of all subjects were decreased after the intervention period (Table 1). Mean CFQ 11 scores significantly decreased, from 25.3 ± 5.5 to 17.0 ± 5.8 (*p* < 0.01). For comparison, the CFQ 11 scores at their first visit to our hospital (26.4 ± 3.9) are also shown in Table 1. The scores were almost the same between their first visit and the pre-intervention visit, except for patients 3 and 5. The condition of patient 3 had worsened since her first visit to the hospital, as reflected in the changes in her CFQ 11 scores. She began to participate in the recumbent isometric yoga program 2 years after her first visit. In contrast, the condition of patient 5 gradually improved after taking medications such as hochuekkito. However, the improvement was not sufficient to enable her to perform housework or full parenting activities, even one and a half years after the initial visit. Therefore, she chose to participate in the recumbent isometric yoga program. When considering her condition, her CFQ 11 scores before and after the intervention period seemed extremely low. When she was later questioned as to the reason, we found that it was due to her misunderstanding of the word “than usual” in the questionnaire. Whereas the intent was to compare her current condition to when she last felt well, she had been sick and almost bed-bound for some years so she misunderstood “than usual” as “than the days sick in bed” because it had become a regular part of life for her. Because she felt that her symptoms became “less than” or “no more than” her “usual” sick days after practicing recumbent isometric yoga, her score was 5 after the intervention.

### Changes in serum miRNA expression after practicing isometric yoga

After practicing recumbent isometric yoga for 3 months, four miRNAs were found to be significantly upregulated (Table 2) and 42 were significantly downregulated (Table 3). The most upregulated miRNA was *Homo sapiens* (hsa)-miR-668-3p (*p* < 0.01) and the second was hsa-miR-19a-3p (*p* = 0.01). Conversely, the most downregulated miRNA was hsa-miR-4723-5p (*p* = 0.01) and the second was hsa-miR-1273 g-3p (*p* = 0.03).

**Table 2:** Upregulated miRNAs after practicing isometric yoga

miRNAs	Ratio (post/pre)	*p*-value
hsa-miR-668-3p	2.48	< 0.01
hsa-miR-19a-3p	2.16	0.01
hsa-miR-4436	2.16	0.05
hsa-miR-485-3p	1.77	0.04

**Table 3:** Downregulated miRNAs after practicing isometric yoga

miRNAs	Ratio (post/pre)	*p*-value
hsa-miR-4723-5p	0.54	0.01
hsa-miR-1273 g-3p	0.56	0.03
hsa-miR-7151-3p	0.57	0.04
hsa-miR-6756-3p	0.57	0.03
hsa-miR-619-5p	0.58	0.02
hsa-miR-6750-3p	0.58	0.03
hsa-miR-940	0.58	0.05
hsa-miR-4507	0.58	0.03
hsa-miR-6741-3p	0.58	0.02
hsa-miR-2467-3p	0.59	0.01
hsa-miR-6880-3p	0.59	0.03
hsa-miR-6804-5p	0.59	0.01
hsa-miR-6759-3p	0.59	0.01
hsa-miR-4478	0.60	0.02
hsa-miR-4279	0.60	0.04
hsa-miR-5001-5p	0.60	0.05
hsa-miR-4515	0.61	0.04
hsa-miR-665	0.61	0.03
hsa-miR-7111-5p	0.61	0.03
hsa-miR-4695-5p	0.61	0.02
hsa-miR-675-5p	0.61	0.03
hsa-miR-4525	0.61	0.04
hsa-miR-3154	0.61	0.02
hsa-miR-4750-5p	0.63	0.03
hsa-miR-6749-3p	0.63	0.05
hsa-miR-1236-5p	0.63	0.01
hsa-miR-1306-5p	0.64	0.04
hsa-miR-8485	0.64	0.02
hsa-miR-4505	0.64	0.05
hsa-miR-6886-3p	0.64	0.04
hsa-miR-6754-5p	0.64	0.02
hsa-miR-4723-3p	0.64	0.01
hsa-miR-6086	0.64	0.01
hsa-miR-1260a	0.65	0.05
hsa-miR-4300	0.65	0.04
hsa-miR-4763-3p	0.65	0.02
hsa-miR-4428	0.66	0.03
hsa-miR-4739	0.66	0.05
hsa-miR-4769-3p	0.66	0.01
hsa-miR-6820-5p	0.66	0.04
hsa-miR-939-5p	0.66	0.04
hsa-miR-6792-3p	0.66	0.04

## Discussion

To our knowledge, this is the first report to suggest that recumbent isometric yoga alters the serum miRNAs of patients with ME/CFS. We demonstrated that regular practice of recumbent isometric yoga reduced the CFQ 11 scores of patients with ME/CFS. Several studies have already suggested the beneficial effects of yoga, although the type of yoga was different from that used in our studies, in at least a subset of patients with ME/CFS [4–6, 19] [46]. In a case report of one patient with CFS, yoga-based lifestyle intervention improved their clinical profile, positive aspects of personality, and subjective well-being, in addition to reducing anxiety [4]. In our previous study we conducted a randomized, controlled trial to assess the effect of isometric yoga on patients with ME/CFS and found that isometric yoga practiced in a seated position improved fatigue and pain in conventional treatment-resistant patients with ME/CFS who were able to sit for at least 30 min [5]. We also developed a recumbent isometric yoga program for patients who found it difficult to sit for long periods of time and were reluctant to practice seated isometric yoga. We found that practicing recumbent yoga for 3 months decreased the CFQ 11 scores of these patients [6]. Participants in the present study were a different cohort than those in our previous study [6]; however, we obtained the same results. These studies suggest that regular practice of recumbent isometric yoga reduces the fatigue of patients with ME/CFS. However, the present results should be interpreted carefully because, as with patient 5, it is possible that the questionnaire may have been misunderstood. Furthermore, as was previously pointed out [47], when evaluating the changes of patients with a high score (near 33), the potential for a ceiling effect must also be taken into consideration.

The present study also demonstrated changes in several serum miRNA levels of patients with ME/CFS after practicing recumbent isometric yoga for 3 months. Serum miRNAs, which are known to be stably encapsulated in vesicles and secreted or excreted from cells or tissues, have been proposed as clinical biomarkers, such as in cancer detection [31–33]. In the present study, we found that 4 miRNAs were upregulated, and 42 miRNAs were downregulated in serum samples after practicing recumbent isometric yoga. However, these miRNAs do not correspond with the miRNAs reported to be differentially expressed in patients with ME/CFS compared to healthy subjects. For example, Brenu et al. demonstrated that plasma levels of miR-127-3p, miR-142-5p and miR143-3p were upregulated in patients with ME/CFS in comparison to non-fatigued control subjects [24]. They also found that miR-21 and several other miRNAs were decreased in the natural killer (NK) and CD8^+^ T cells of ME/CFS patients [23]. Another study reported that 34 miRNAs were upregulated in peripheral blood mononuclear cells of patients with ME/CFS, and that four miRNAs, miR-99b, miR-330, miR-126, and miR-30c, may be useful biomarkers of ME/CFS [25]. Our study failed to replicate the results of these previous studies in that we did not observe changes in these miRNAs after recumbent isometric yoga. Therefore, we must be cautious in our interpretation of the miRNAs that were changed after recumbent isometric yoga and whether they actually affect the pathophysiological mechanisms of ME/CFS.

The reasons for this discrepancy are unclear. However, there are several possible explanations. One is that isometric yoga improves fatigue not by reversing the mechanisms that are involved in the development of ME/CFS but by acting on other mechanisms, e.g. enhancing fatigue-relieving mechanisms, which are not directly responsible for the pathophysiology of ME/CFS. Another reason could be related to the different sample types that were used in the different studies [23–25]. These various sample types, i.e. plasma, serum or NK cells, could have different miRNA profiles, thus the findings might not be readily comparable. Additionally, the observed differences might also result from varied dietary habits. Recent studies have reported that different dietary habits affect the plasma miRNA expression of healthy individuals [48]. Because this study was conducted in Japan and previous studies were conducted in Australia [23, 24] and the United Kingdom [25], it is possible that Japanese dietary habits affected the outcomes.

Among the upregulated miRNAs, miR-19a, the second most upregulated miRNA, is of particular interest because it is known to target TNF-α [49]. Although the findings are inconsistent [21, 22], immunological abnormalities, including increased TNF-α [50], have been documented in patients with ME/CFS [50]. In general, practicing yoga is known to shift autonomic nervous system function from a sympathetic nerve-predominant state to a parasympathetic nerve-predominant state and to reduce inflammatory markers, such as TNF-α, interleukin-1 (IL-1), or nuclear factor kappa B (NF-κB), as well as reduce stress hormones [51–53]. Increased TNF-α and IL-1 are reported to be correlated with fatigue, sadness, autonomic symptoms, and a flu-like malaise in these patients [18]. We previously observed that seated isometric yoga significantly reduced the TNF-α serum level [19]. Therefore, the upregulation of miR-19a expression by isometric yoga might be associated with a reduction of fatigue through the decreased production of TNF-α. Additionally, yoga has been shown to reduce methylation of the TNF region of DNA in chronically stressed women [54]. Therefore, yoga-induced changes in DNA methylation might also contribute to a decrease in TNF-α. Furthermore, vagal nerve activation by yoga [19], which is impaired in ME/CFS patients [55, 56], might also contribute to a decrease in the serum TNF-α level via vagal anti-inflammatory pathways [57].

miR-485 [58] as well as miR-19a [59] have inhibitory effects on TGF-β signaling and the expression levels of TGF-β1. Several studies, but not all [60], have reported that the blood level of TGF-β1 of patients with ME/CFS was higher than in healthy subjects [16]. We have previously observed a correlation between the reduction in fatigue by seated isometric yoga with a reduction in the plasma level of TGF-β1 [39]. Therefore, miR-485 might play a beneficial role via the inhibition of TGF-β signaling. The role of miR-668, which was the most upregulated after yoga, in ME/CFS remains uncertain. Previous studies have indicated that it plays a protective role in acute kidney injury by repressing mitochondrial fission-associated protein [61, 62] and enhances resistance in breast cancer cells [63].

In contrast to the upregulated miRNAs, miR-4273, which was the most downregulated in this study, has been shown to inhibit prostate cancer growth through regulation of Abelson kinase [64]. miR-940, another downregulated miRNA, can promote angiogenic abilities of cerebral microvascular endothelial cells after cerebral infarction [65] and induce osteoblastic phenotypes in the bone metastatic microenvironment [66]. Although the functions of most of the downregulated miRNAs remain unknown, these miRNAs could also be related to the fatigue-relieving mechanisms in ME/CFS.

There are several limitations in the present study. First, we were unable to blind the treatment and did not have a control group for comparison. Because miRNA expression can change due to fluctuations in lifestyle factors such as diet [48], exercise [67], or sleep [68, 69], it is undeniable that the present results could be affected by these or other unknown factors. Second, we did not perform corrections for multiple comparisons. Therefore, the data in the current study should be considered exploratory pilot data. Third, the sample size was small. Because it was not certain if recumbent isometric yoga affects circulating miRNAs, this study was conducted as a pilot study to assess if recumbent isometric yoga actually affects circulating miRNAs. Because this study suggests that isometric yoga can change the serum miRNA level, future studies should attempt to replicate the present results with a larger sample size. Fourth, we did not validate the expression of miRNAs using quantitative real-time polymerase chain reaction. Therefore, the possibility remains that there may be slight deviations from the expression levels observed in the blood. Finally, we could not investigate the actual targets of the affected miRNAs in this clinical study. To identify the targets of these miRNAs, further basic research is needed. There is, of course, a limitation on using the CFQ 11, a subjective measure, to evaluate fatigue because it is possible that the scores could be affected by an expectancy effect or a ceiling effect. Therefore, future studies should include objective measures in addition to the CFQ 11 to assess the fatigue-relieving effects of recumbent isometric yoga.

Despite these limitations, this is the first study to show that isometric yoga impacts the expression of miRNAs. It also suggests that these altered miRNAs may have roles in improving ME/CFS and could represent biomarkers for improvement of ME/CFS symptoms by isometric yoga.

## Conclusions

Regular practice of recumbent isometric yoga was associated with changes in the serum expression of miRNAs in patients with ME/CFS. It is possible that these miRNAs are associated with the symptom-relieving effects of isometric yoga on ME/CFS and that circulating miRNAs might be promising biomarkers for the fatigue-relieving effects of recumbent isometric yoga.

## Data Availability

Data sharing is not applicable.

## Abbreviations

CFS: Chronic fatigue syndrome
DHEA-S: Dehydroepiandrosterone sulfate
FS: Fatigue scale
has: *Homo sapiens*
IL: Interleukin
ME: Myalgic encephalomyelitis
miRNA: microribonucleic acid
mRNA: messenger ribonucleic acid
NF-κB: Nuclear factor kappa B
SD: Standard deviation
TGF: Transforming growth factor
TNF: Tumor necrosis factor
